# Organ and tissue donation in clinical settings: a systematic review of the impact of interventions aimed at health professionals

**DOI:** 10.1186/2047-1440-3-8

**Published:** 2014-03-14

**Authors:** Frédéric Douville, Gaston Godin, Lydi-Anne Vézina-Im

**Affiliations:** 1 Institut universitaire de cardiologie et de pneumologie de Québec, 2725, chemin Sainte-Foy, Room Y-3495, Quebec, (Quebec) G1V 4G5, Canada; 2 Ferdinand-Vandry Building, Faculty of Nursing, Laval University, 1050, avenue de la médicine, Quebec, (Quebec) G1K 7P4, Canada

**Keywords:** tissue and organ procurement, health professional, program development, professional education, hospital

## Abstract

In countries where presumed consent for organ donation does not apply, health professionals (HP) are key players for identifying donors and obtaining their consent. This systematic review was designed to verify the efficacy of interventions aimed at HPs to promote organ and tissue donation in clinical settings. CINAHL (1982 to 2012), COCHRANE LIBRARY, EMBASE (1974 to 2012), MEDLINE (1966 to 2012), PsycINFO (1960 to 2012), and ProQuest Dissertations and Theses were searched for papers published in French or English until September 2012. Studies were considered if they met the following criteria: aimed at improving HPs’ practices regarding the donation process or at increasing donation rates; HPs working in clinical settings; and interventions with a control group or pre-post assessments. Intervention behavioral change techniques were analyzed using a validated taxonomy. A risk ratio was computed for each study having a control group. A total of 15 studies were identified, of which only 5 had a control group. Interventions were either educational, organizational or a combination of both, and had a weak theoretical basis. The most common behavior change technique was providing instruction. Two sets of interventions showed a significant risk ratio. However, most studies did not report the information needed to compute their efficacy. Therefore, interventions aimed at improving the donation process or at increasing donation rates should be based on sound theoretical frameworks. They would benefit from more rigorous evaluation methods to ensure good knowledge translation and appropriate organizational decisions to improve professional practices.

## Review

### Background

The number of patients awaiting organ or tissue transplantation continues to grow throughout the world [1-4]. The shortage of organ and tissue donors is widely studied and several factors explaining why individuals accept or refuse to consent to organ and tissue donation are reported in the literature [5]. Simpkin *et al*. [6] conducted a review of modifiable factors that influence relatives’ decisions to allow organ donation. This review suggests that the skills of individuals making the request to donate may have a significant impact on consent rates. Based on this information, evaluating the efficacy of interventions among HPs to increase donation seems relevant.

The donation process depends on potential donor identification and on HPs approaching families for donation consent. Since HPs are responsible for this approach to families, they are the gatekeepers for organ and tissue donor notification.

Consent to organ and tissue donation is the end point resulting from many actions undertaken by HPs (from identifying potential donors to referring donors to an organ and tissue procurement representative). In fact, many of these actions can be viewed as professional practices and as forms of human behavior. Thus, interventions should take advantage of behavioral theories and behavior change strategies in their design [7-11]. Past studies have demonstrated the importance of developing theory-based interventions in order to enhance their potential success in changing behavior [12,13]. The absence of theoretical bases for interventions and the selection of appropriate behavioral change techniques are two of the main problems in behavior change research projects [14-17]. Grimshaw *et al*. [15] suggest exploring the applicability of behavioral theories to the understanding of behavior change among HPs.

Several systematic reviews on organ donation have been published. These systematic reviews have cover different aspects of organ donation including the factors influencing families consent to donation [6], the attitude of the public towards living donors [18], the educational interventions offered in high schools [19], the management of donor brain death [20] and professional’s attitude regarding the heart-beating donors [21]. However, there is no systematic review on the efficacy of interventions among HPs to encourage them to approach families for consent or increasing donation rates. This is an important aspect of organ donation because donor identification and obtaining the consent of family are necessary conditions to the donation process.

This systematic review was designed to identify and analyze the impact of interventions aimed at HPs to improve donation-promoting professional practices in clinical settings. Secondary outcomes consisted of verifying whether such interventions were effective in improving donation rates and exploring associated behavior change strategies and the underlying theoretical framework.

## Methods

### Search strategy

The most relevant electronic databases covering the field of behavior change among HPs are those in health and psychology. CINAHL (1982–2012), COCHRANE LIBRARY (Cochrane Reviews, Other Reviews, Trials, Methods Studies, Technology Assessments, Economic Evaluations, Cochrane Groups), EMBASE (1974–2012), MEDLINE (1966–2012), PsycINFO (1960–2012), and ProQuest Dissertations and Theses were searched for papers published in French or English until September 2012.

The search strategy included the following concepts: 1) health professionals; 2) organ and tissue donation; and 3) interventions or strategies. This search strategy was adapted according to the terminology of the various databases. Moreover, bibliographies of potential studies were analyzed manually to find other key words relevant to the search strategy and studies not identified with the main search strategy. Only French and English papers were considered for review for practical reasons. The complete search strategy for each database is presented in Additional file 1.

### Study eligibility criteria

To be eligible for inclusion, studies had to adopt an experimental or quasi-experimental design reporting interventions aimed at HPs in clinical settings in order to improve their practices regarding the donation process or to increase the donation rates. They also had to report behavioral measures of the donation process or impact on organ and tissue donation rates as the study outcome.

In this study, HPs refer to professionals with medical training whose jobs require them to be in contact with patients and who are in a position to ask for donor consent. The concept of HP includes family physicians, specialist physicians, nurses or any other allied HPs who meet families in their daily practice. It also includes physicians in training (residents or interns), but excludes healthcare students and administrators not in contact with patients.

Also, the interventions had to be offered to HPs with the intention of modifying their practice regarding the donation process or at increasing donation rates. Such interventions could take the form of educational (for example, flyers, workshop, or lecture) [22,23], organizational (for example, hospital personnel structure change, or guidelines) [24], or regulatory strategies. These interventions or strategies were retained insofar as they were aimed at HPs caring for patients.

From a methodological point of view, the studies had to include a control group. However, to ensure that the study would not overlook relevant interventions that might have been effective, intervention studies without a control group, but with a pre-post analysis, were considered in a separate analysis.

Finally, to be included in the review, the intervention outcome had to be reported as a behavioral measure of the donation process (objective or self-reported), based on Kirkpatrick’s third level of program evaluation [25], or as the impact on organ and tissue donation rates. Behavioral measures could be a specific action (behavior) in the donation process, such as identifying a potential donor, approaching families to initiate discussion, obtaining signed consent for a donation or referring a potential donor to an organ and tissue donor representative. Articles reporting the impact on organ and tissue donation rates were considered even if the study did not assess behavioral outcomes to ensure comprehensiveness of the interventions reported in this review.

Studies that did not include HPs were excluded, as were those not directly aimed at changing HPs’ behavior, such as the implementation of an Organ Procurement Organization (OPO) coordinator in a hospital. Although one of the OPO’s duties involves identifying potential donors and approaching families to initiate donation discussion, their implementation could not be considered as an intervention intended for HPs (nurses and physicians) to modify their practices regarding the donation process; the latter would still have to notify the OPO and procurement organizations of potential organ and tissue donors.

Finally, studies concerning HPs’ reactions following an intervention or their level of knowledge following the intervention [25] were not considered if the assessed outcomes did not include the HPs’ behavior or donation rate.

Sorting of the studies by their titles and abstracts was first carried out by FD in order to select the articles meeting the inclusion criteria. Thereafter, the full text articles that met the inclusion criteria were screened independently by FD and LAVI, and decisions were compared.

### Study quality assessment

Quality assessment of the studies was performed using criteria inspired by Morrison [26] and Reed [27], who recommend questions for appraising reports of medical education interventions.

Three criteria were selected to assess the population (randomized sample; justification of sample size and existence of a control group). Two criteria evaluated the intervention (allocation concealment and theory underlying the intervention). Two criteria appraised the assessment tool (validity and reliability). Finally, two criteria assessed the statistical approach used (intention-to-treat) and the level of attrition at follow-up.

No assessment for the risk of bias across studies was performed because the interventions had different objectives, populations and outcomes, making it impossible to obtain cumulative evidence.

### Data extraction

A first coding was carried out on one study to verify if there was agreement on the extraction of data and to confirm the quality of the coding sheet. In case of disagreement between the two reviewers, the final decision was taken after discussion with a third reviewer (GG).

The following data were extracted from the selected studies: authors, year of publication, population under study and sample size. The study data were extracted according to the recommendations for evaluating educational interventions [26,27]. Thus, the reported variables were: objective of the study; intervention type (educational or organizational) and strategy; duration of follow-up; behavior change techniques; and study methodology, outcomes and results. The theory underlying each intervention was also extracted.

To help classify HPs’ strategies and relate those to the most recognized and effective theory-based strategies, behavior change techniques were analyzed using the taxonomy developed by Abraham and Michie as reference [11]. This taxonomy contains 26 behavior change techniques used in interventions based on behavior change theories such as the theory of reasoned action [28], the theory of planned behavior [29], the social cognitive theory [30], the information-motivation-behavioral skills models [31] and other behavior change theories.

### Data analyses

Based on the studies retained, a descriptive analysis of selected studies (study objective; intervention type (educational or organizational) and strategy adopted; duration of the follow-up; behavior change techniques used; and study methodology, outcomes and results) was completed prior to identifying effective interventions. Interventions with a control group and interventions with a pre-post analysis are described separately.

A risk ratio was calculated for each outcome among the studies with a control group. The risk ratio was determined based on the number of participants in each group (experimental and control) and on the frequency of HPs’ behavior adoption. Thus, the analysis allowed the identification of significant differences between the two groups following the implementation of an intervention.

## Results

### Review statistics

**Figure 1:**
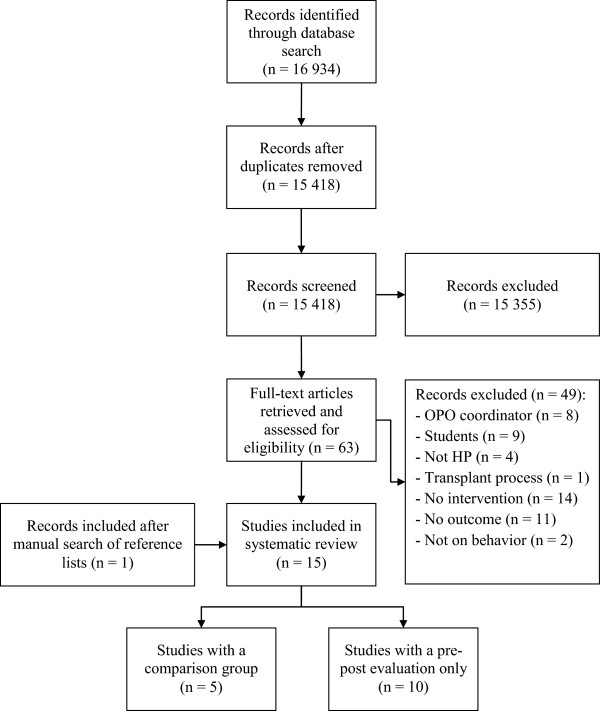
Flow chart diagram.

A total of 15 studies assessing interventions among HPs in clinical settings aimed at improving professional practices regarding the donation process or increasing donation rates were identified. The results of the search strategy are presented in Figure 1. All studies included used educational, organizational or a combination of both types of interventions to promote professional practices regarding the donation process. These took the form of in-service meetings, workshops, conferences, print documents, examples provided of situations associated with the organ and tissue donation process and identification of donation criteria or information on how to approach a potential donor [23,32,33].

### Study quality assessment

The 15 studies were assessed regarding population and the intervention assessment tool. In general, study quality was low. No study used a randomized population or justified their sample size. Only five studies used a control group. Allocation concealment of the intervention was neither relevant nor mentioned for all the studies included, and 14 of the 15 studies did not use a theory-based intervention. Where relevant, the validity and reliability of the assessment tools were not mentioned. Among the studies with a control group, there was no intention-to-treat analysis. Finally, the attrition rate was appropriately mentioned when required. The results of the quality assessment for the studies of the present review are available in Table 1.

**Table 1:** Summary of quality assessment for the studies included

	**Population**			**Intervention**		**Assessment tool(s)**^**a**^		**Analysis**	
**Papers**	**Randomization**	**Justification of sample size**	**Control group**	**Allocation concealment**	**Underlying theory**	**Validity**	**Reliability**	**Intention-to-treat**	**Attrition rate**
Alonso, Fernandez, Mataix *et al*. (1999)	No	Not mentioned	No	N/A	None	N/A	N/A	N/A	N/A
Beasley, Capossela, Brigham, Gunderson and Gortmaker (1997)	No	No	No	N/A	None	N/A	N/A	N/A	N/A
Bleakley (2010)	No	No	No	N/A	None	N/A	N/A	N/A	N/A
Dettle, Sagel and Chrysler (1994)	No	No	Yes (but no statistical comparison between groups)	Not mentioned	None	Not mentioned	Not mentioned	no	40% attrition;
									No analysis of dropouts
Kittur, McMenamin and Knott (1990)	No	No	Yes	Not randomly assigned	None	N/A	N/A	N/A	N/A
Light (1987)	No	No	Yes (but no statistical comparison between groups)	Not randomly assigned	None	N/A	N/A	N/A	N/A
Milanés, Gonzalez, Hernandez, Arminio, Clesca and Rivas-Vetencourt (2003)	No	No	No	N/A	None	N/A	N/A	N/A	N/A
Nelson, Marymont, Durand, Reyes and Davis (1992)	Random cluster probability method	No	Yes	Not randomly assigned	None	Field testing of the questionnaire (but validity/reliability assessment not detailed)		N/A	N/A
Niday, Painter, Peak *et al*. (2007)	No	No	No	N/A	None	N/A	N/A	N/A	N/A
Riker and White (1995)	No	No	Yes	Not randomly assigned	None	N/A	N/A	N/A	N/A
Shafer, Durand, Hueneke, *et al*. (1998)	No	No	No	N/A	None	N/A	N/A	N/A	N/A
Stark, Wikoren and Martone (1994)	No	No	No	N/A	None	N/A	N/A	N/A	N/A
Taylor, Young and Kneteman (1997)	No	No	No	N/A	‘Change theory’ (not referenced)	N/A	N/A	N/A	N/A
Van Gelder, Van Hees, de Roey, Monbaliu, Aerts, Coosemans *et al*. (2006)	No	No	No	N/A	None	N/A	N/A	N/A	N/A
Wight, Cohen, Roels and Miranda (2000)	No	No	No	N/A	None	N/A	N/A	N/A	N/A

^a^The assessment tools assessed were only those regarding outcomes assessed in this systematic review, that is, professional practices or donation rates; when the outcome was an objective measure (donation rate or any quantitative item retrieved from medical records review), validity and reliability were considered nonapplicable.

N/A, not applicable.

### Efficacy of the interventions

#### Intervention studies with comparison groups

Among the 15 studies included, only five had a comparison group (Table 2) [23,32-35]. The specific populations in these studies were nurses [32-34], physicians [23,33,34] and residents in medicine [35]. In addition to HPs, three studies also included other allied HPs such as chaplains or administrators [32-34]. All the studies used educational interventions to increase donation and one also used an organizational strategy. None of these interventions were based on a theoretical framework. According to the list of behavior change techniques [11], the majority of the strategies provided instruction on the donation process, the HPs’ role or how to cope with families’ reactions.

**Table 2:** Description of the interventions on organ and tissue donation with comparison groups

**Authors (year); country**	**Purposes**	**Populations (*n*)**	**Interventions**	**Follow-up**	**Behavior change technique**	**Study methodology**	**Outcomes**	**Results**
								**(Experimental versus Control)**
Dettle, Sagel and Chrysler (1994); United States	To gain a better understanding of health care professionals’ experience, knowledge, attitudes, and comfort level regarding organ and tissue donation	Nurses and Chaplains (*n* = 343)	Educational:	6 months	• Provide instruction	Health professionals survey	Approached family	Experimental 18% → 38% (*P* = .039)
			• Formal in-service on organ and tissue donation					
								Control 4% → 25% (*P* < .001)
			• Unit meeting addressing donation issues					
			• Dealing with a family of an actual donor					
Kittur, McMenamin and Knott (1990); United States	To evaluate the impact of an organ donor and tissue donor advocacy program on community hospitals	Hospital staff: physicians, nurses and administrators (*n* = not mentioned)	Educational:	12 months	• Provide instruction	Not mentioned	Referred potential donor	44 donors versus 2 donors
			• Hospital’s organ and tissue donation policies and procedures		• Provide contingent rewards			
					• Teach to use prompts or cues			
							Organ and/or tissue donor recovered	18 donors versus 1 donor
			• Sending letter of gratitude to requestors					
			• Sending letter reminding to request all eligible patients					
			Organizational:					
			• Developing a donor advocate role					
Light (1987); United States	To evaluate the efficacy of including printed criteria and procedures with the autopsy permits as a simple, inexpensive method of increasing cornea donation	Residents (*n* = 84)	Educational:	4 months	• Provide information on consequence	Eye bank data analysis	Organ and/or tissue donor recovered	Experimental 1.8% → 10.2% (*P* = .009)
			• Instruction for cornea donation and a checklist of donation procedures		• Provide instruction			
								Control 7.1% → 8.5% (not significant)
Nelson, Marymont, Durand, Reyes and Davis (1992); United States	To examine the organ procurement organization’s educational activities and their effects on attitudes, knowledge, and referral behavior	Nurses, physicians and chaplains (*n* = 265)	Educational:	Not mentioned	• Intervention not described	Health professionals survey	Approached family	59% versus 46% (*P* = .027)
			• Continuing medical education					
			• Newsletters					
			• Other publications					
			• Requestor’s workshop					
			• In-service training session					
			• Others programs					
							Referred potential donor	46% versus 9% (*P* = .001)
Riker and White (1995); United States	To evaluate physician response to an educational program to increase referral of potential organ or issue donors in an emergency department	Physicians (*n* = not mentioned)	Educational:	6 months	• Provide instruction	Hospital charts review	Approached family	65% versus 6.6% (*P =* .001)
			• One-hour conference on the physician’s role in requesting donation and review the criteria for donation and services available from transplant program					
							Obtained donation consent	32% versus 6.6% (*P* = .08)
							Organ and/or tissue donor recovered	48% versus 5.5% (*P =* .003)

Relative risks (risk ratios) were computed to determine how likely participants were to adopt a behavior related to organ and tissue donation following an intervention, compared with those not exposed to the intervention (Table 3). Due to a high level of heterogeneity, the relative risks were calculated independently for each study and not pooled together.

**Table 3:** Efficacy of interventions with a comparison group on health professionals’ (HPs) behavior

**Studies**	**Outcomes**	**Risk ratio (95% CI)**
Dettle *et al*. (1994)	Approached family	1.53 (0.82, 2.85)
Kittur *et al*. (1990)	Referred potential donor	N/A
	Organ and/or tissue donor recovered	N/A
Light (1987)	Organ and/or tissue donor recovered	1.19 (0.45, 3.12)
Nelson *et al*. (1992)	Approached family	1.28 (1.01, 1.61)
	Referred potential donor	5.04 (2.79, 9.10)
Riker and White (1995)	Approached family	9.71 (1.44, 65.53)
	Obtained donation consent	4.85 (0.69, 34.28)
	Organ and/or tissue donor recovered	8.67 (1.24, 60.58)

The intervention studies of Nelson *et al*. [33] and Riker and White [23] showed significant relative risks for the following: approaching families [23,33], referring potential donors [33] and increasing donation rates [23]. However, the interventions of Dettle *et al*. [32], Light [35] and Riker and White [23] did not result in a significant increase in the number of signed consents for donation. No relative risk could be computed for the interventions of Kittur *et al*. [34], since the results were presented in absolute numbers instead of percentages, and there were no data on the total size of the groups.

#### Intervention studies without a comparison group (pre-post assessments)

The remaining ten studies used pre-post assessments (Table 4) [36-45]. These studies evaluated behavior change toward donation among HPs or the impact of their intervention on donation rates. The participants targeted in these interventions were mainly nurses and physicians. However, six of these studies involved hospital staff, without specifying which types of HPs were targeted [36,38,40,42,44,45]. Also, in six of the ten studies, the number of participants was not provided [36,37,41-44].

**Table 4:** Description of interventions on organ and tissue donation with only pre-post assessments

**Authors (year); country**	**Purposes**	**Populations (*n*)**	**Interventions**	**Follow-up**	**Behavior change technique**	**Study methodology**	**Outcomes**	**Results**
								**(pre → post)**
Alonso, Fernandez, Mataix *et al*. (1999); Spain	To present the results of a pilot study carried out in Seville, Spain, evaluating the donor action program	Hospital staff (*n* = not mentioned)	Educational:	12 months	• Provide instruction	Medical records review	Detected potential donor	81.0% → 97.5%
			• Training in family interview and communication					
			• Training in donor detection and brain death diagnosis				Organ and/or tissue donor recovered	32.1% → 44.4%
			• Creating guidelines for donation process					
Beasley, Capossela, Brigham, Gunderson and Gortmaker (1997); United States	To increase organ donation in 50 hospitals in three organ procurement organization service areas simultaneously by using a large-scale intervention	Physicians, residents, nurses, social workers, chaplains and administrators (*n* = not mentioned)	Educational:	24 months	• Provide instruction	Medical records review	Approached family	69.0% → 85.6% (*P =* .001)
			• Presentation of donation protocols		• Provide feedback on performance			
			• Review health professional role in donation process				Referred potential donor	55.5% → 80.2% (*P =* .001)
			• Department meeting				Obtained donation consent	50.9% → 52.2% (not significant)
			• In-services					
			Organizational:				Organ and/or tissue donor recovered	32.9% → 42.5% (*P =* .005)
			• Organ donation protocols					
			o Potential donor identification					
			o Notification of the organ procurement organization					
			o Ensuring decoupled request					
			o Private setting to ask for donation					
			o Active inclusion of organ procurement organization in request					
Bleakley (2010); United Kingdom	To increase the number of donated organs through an effective donor identification and referral scheme in a large acute hospital’s critical care units	Clinical staff	Educational:	12 months	• Intervention not described	Not mentioned	Referred potential donor	4 → 121 (donors)
		(*n =* 170)	• Education program on required referral					
			Organizational:					
			• Hospital policy on how to make a referral					
Milanés, Gonzalez, Hernandez, Arminio, Clesca and Rivas-Vetencourt (2003); Venezuela	To find solutions to the critical donor shortage situation, and its negative socioeconomic impact in our society, by implementing a transplant coordination program in a hospital with a variety of departments, including neurosurgery and kidney transplantation	Healthcare staff in the critical care area (*n* = 97)	Educational:	24 months	• Provide instruction	Medical records review	Detected potential donor	8.1% → 57.5%
			• Detection, identification and donor criteria					
			• Death diagnostic					
			• Donor maintenance				Organ and/or tissue donor recovered	1.6% → 9.1%
			• Organ and tissue viability studies					
			• Family interview, requesting consent					
			• Organ sharing, allocation and preservation					
			• Transplant ethics and legislation					
Niday, Painter, Peak *et al*. (2007); United States	To implement and evaluate a scripted information about organ and tissue donation for hospice inpatient on admission	Nurses	Educational:	6 months	• Provide instruction	Review of death records	Organ and/or tissue donor recovered (corneal rates)	6.3% → 20.6%
		(*n* = 12)	• Scripted instruction to prompt nurses to introduce the subject of donation					
							Organ and/or tissue donor recovered (tissue rates)	0.0% → 0.0%
			Organizational:					
			• Give tissue donation information upon admission as part of the normal admission process and then repeated at the time of death.					
Shafer, Durand, Hueneke, *et al*. (1998); United States	To determine whether donors could be produced from non-donor hospitals	Nurses and hospital staff	Educational:	17 months	• Intervention not described	Monthly death records audit	Referred potential donor (organ)	24 → 139 (donors)
		(*n* = 25 hospitals)	• Training activities					
			• Education programs and materials				Referred potential donor (tissue)	202 → 3,931 (donors)
			Organizational:					
			• Develop in-house coordinators					
							Organ donor recovered	8 → 44 (donors)
							Tissue donor recovered	154 → 423 (donors)
Stark, Wikoren and Martone (1994); United States	To develop and pilot an organ donation program that focuses on the collaborative efforts of the entire health care team, hospital administration and organ procurement agency	Physicians, nurses and hospital personnel (*n* = not mentioned)	Educational:	24 months	• Provide information on consequences	Not mentioned	Detected potential donor	45.7% → 92.0%
			• Partners in organ donation program					
			o Promote positive attitudes toward donation (awareness)		• Provide instruction		Obtained donation consent	17.1% → 56.0%
			o Recognize potential donor					
			o Offering the option of donation					
			o Support the grieving of donor families					
			Organizational:					
			• Develop nurse requestor role					
Taylor, Young and Kneteman (1997); Canada	To describe the development of a program to cross-train critical care nurses as organ procurement coordinators	Intensive care units nurses (*n* = not mentioned)	Educational:	Not mentioned	• Provide instruction	Not mentioned	Donation rates	18 donors per million population → 31 donors per million population (72% increase rate)
			• Classroom instruction					
			• Preceptor clinical experience					
Van Gelder, Van Hees, de Roey, Monbaliu, Aerts, Coosemans *et al*. (2006); Belgium	To measure the impact of an intervention plan designed to optimize the donor detection process and donor referral patterns	Departments of neurology, neurosurgery, anesthesiology, intensive care medicine and abdominal transplant (*n* = not mentioned)	Educational:	48 months	• Provide instruction	Not mentioned	Organ and/or tissue donor recovered	230 → 301 (donors) (*P* < .05)
					• Provide feedback on performance			
			• Information on donor criteria					
			• Communication between donor and transplant centers					
			Organizational:					
			• Facilitation of procedure					
							Tissue donor recovered	66 → 180 (donors) (*P* < .001)
Wight, Cohen, Roels and Miranda (2000); United Kingdom	To evaluate the immediate (6 months), short-term (1 year) and sustained (2 years) effects of the Donor Action program on donation rates in different countries	Intensive care units staff (*n* = not mentioned)	Educational:	12 months (United Kingdom)	• Provide instruction	Medical records review	Organ donor recovered (United Kingdom.)	122% increase (6 months)
			• Educational program on:	24 months (Spain)	• Provide feedback on performance			
			o Family care and communication					
								40% increase (12 months)
			o Donor maintenance		• Prompt practice			
			o Organ retrieval					
			Organizational:					
			• Forming a Donor Action committee					
							Have referred potential donor (Spain)	16% increase (24 months)
							Organ donor recovered (Spain)	33% increase (24 months)

All the studies used educational strategies or a combination of organizational and educational strategies to promote donation behavior among HPs. In the study of Taylor *et al*. [41], there were references to the concept of change theory in the development of their intervention, but none of the other studies used a theoretical framework to guide the development of their intervention. The most common technique was to provide instruction on the donation process, the identification of donor criteria, the HPs’ role in the donation process and how to approach family members to initiate discussion.

## Discussion

This systematic review summarized the studies assessing educational and/or organizational interventions aimed at HPs to improve professional practices regarding the donation process or increase donation rates in clinical settings. A total of 15 studies were identified, among which only five had a comparison group. No study referred to a theoretical framework, either for the development of the interventions or their assessment. The behavior change technique most often used consisted of providing instruction on the donation process, including criteria and the role of HPs (how to approach family members, to initiate discussion or how to cope with families’ reactions).

Based on our review, the selected interventions aimed at changing HP practices regarding donation were developed, for the most part, more than a decade ago. Recent developments in donation emphasized the introduction of OPO representatives [46,47] and the regulation ensuring donation after death (such as presumed consent) [1]. If organ donation rates increased following the introduction of OPOs in clinical settings [46,47] or following a change in regulations [1], HPs still have to notify procurement organizations of any potential donors, leaving place for more research and interventions to help HPs in the donation process.

### Impact on donation-promoting professional practices

Although there are many interventions aimed at changing HPs’ behavior toward the organ and tissue donation process in clinical settings, only a few were carried out exclusively among HPs whose job position requires them to be in contact with patients and who are in a position to ask for donation consent [23,32]. Indeed, most of the interventions also targeted hospital administrators, clerical staff and chaplains [32-34,37]. As such, it is difficult to isolate the impact of these interventions on nurses’ and physicians’ behavior.

The lack of studies assessing the behavior changes or health outcomes in this literature review is consistent with a recent publication that reviewed the evaluation of inter-professional education programs. According to Kirkpatrick’s levels, [25] only 9.7% of program evaluations assessed changes in behavior, 0.004% examined organizational practice changes and no items addressed benefits to patients [48]. Similar results were obtained in continuing nursing education programs [49].

### Impact on donation rates

Interestingly, more than half of the studies included used an objective measure of the impact of the intervention on donation rates. This was achieved by extracting the information from medical records to evaluate the number of deaths (potential donors) and the number of actual donors [23,39,44]. This type of measure is obviously better than using self-reported behavior and provides more confidence in the observed effects.

### Behavior change strategies and underlying theoretical framework

Surprisingly, in spite of the HPs’ role of gatekeeper in the donation process, there is a lack of sound theoretical interventions aimed at improving professional practices regarding the donation process or at increasing donation rates. None of the interventions were developed with reference to a behavior change theory, except the study by Taylor, Young and Kneteman [41], which mentioned the use of the concept of change theory, but without explaining how it was applied.

The fact that the interventions included in the present review had a poor theoretical basis and an inappropriate evaluation of their impact has important clinical implications. OPOs and donation stakeholders seem to apply nontheory-based intervention strategies without being sure of their efficacy. These interventions have an important cost for the healthcare system without resulting in significant changes (for example, increases in donation rates).

### Quality of reviewed studies

The interventions presented several weaknesses in their evaluation designs. For instance, only five of the 15 studies identified used a comparison group to ensure that the intervention effects could be attributed to the implemented change strategy [23,32-35]. In addition, significant methodological flaws (for example, vague definition of the intervention, absence of a theoretical framework, lack of explanations on the study design, unjustified sample size) were noted.

Many of the studies included showed nonsignificant improvements in the detection of potential donors, approaching families and achieving consent or increasing donation rates in clinical settings [32,35]. Yet, some studies have proven that providing instruction on the donation process can significantly change HPs’ behavior over a period of 6 to 24 months [23,33]. However, it was not possible to establish whether an intervention was efficient due to methodological flaws, poorly described population or the lack of details on the content of the interventions and evaluation. Moreover, it was not possible to determine the efficacy of studies only using a pre-post evaluation because of the lack of a control group.

### Limitations of the systematic review

The present review has some limitations. Only a small number of studies could be included in the analysis because most did not use a control group to compute a relative risk. Not all interventions reported the required information to compute relative risk (that is, number of participants in the experimental and the control groups). Moreover, the variability of the intervention strategies and the different HP practices on donation prevented the computation of some comparisons and the pooling of relative risks.

## Conclusions

Despite the large number of publications on interventions to improve HPs’ practices regarding the donation process or increase donation rates, few of these interventions have been evaluated, or the associated assessments have methodological flaws that make it difficult to draw clear conclusions regarding their efficacy. Therefore, interventions aimed at improving the donation process or increasing donation rates should be based on sound theoretical frameworks and would benefit from more rigorous evaluation methods to ensure good knowledge translation and appropriate organizational decisions to improve professional practices.

## Abbreviations

HP: health professional; OPO: organ procurement organization.

## Competing interests

The authors declare that they have no competing interests.

## Authors’ contributions

FD contributed substantially to developing and designing the study, acquiring data, analyzing and interpreting data and drafting the manuscript. GG contributed to developing and designing the study, interpreting data and drafting the manuscript. LAVI contributed to extracting data and drafting the manuscript. All authors have read and approved the final manuscript.

## Authors’ information

FD is a PhD candidate at the Faculty of Nursing at Laval University (Quebec City, Canada) and a clinical nurse specialist at the Institut de cardiologie et de pneumologie de Québec. GG is a professor at the Faculty of Nursing at Laval University. LAVI is a research professional at the Faculty of Nursing at Laval University.

## Supplementary Material

Additional file 1 Search strategy for each database. Click here for file
