# Supplementary material for: Organ and tissue donation in clinical settings: a systematic review of the impact of interventions aimed at health professionals
Source: Transplant Res. 2014 Mar 14;3:8. doi: 10.1186/2047-1440-3-8 (PMC4003858; doi:10.1186/2047-1440-3-8)
Supplement: Additional file 1 — Search strategy for each database. [file 2047-1440-3-8-S1.pdf]

Additional file 1

The search strategy for each database

**Cinahl** (1981-2010)
Organ procurement* OR tissue procurement* OR organ donation* OR tissue donation* OR organ donor* OR tissue donor* OR organ transplant* OR tissue transplant* OR eye donation* OR eye donor* OR corneal donor* OR corneal donation* OR organ procurement (Exact major subject heading)<br>AND<br>health care worker* OR health employe* OR health care staff OR health care personnel* OR health care provider* OR health care professional* OR (health AND (team* OR staff* OR practitioner* OR worker* OR manpower) OR nurse* OR physician* OR resident* OR health personnel (Exact major subject heading) OR health manpower (Exact major subject heading) OR students, health occupations (Exact major subject heading)<br>AND<br>information* OR clinical trial* OR dissemination OR guideline* OR protocol* OR policy OR policies OR process* OR recommendation* OR incentive* OR organization* OR administrati* OR optimi* OR improv* OR project* OR workshop campaign* OR poster* OR leaflet* OR session* OR intervention* OR program* OR strategie* OR practice* OR regulation OR trial* OR training* OR implementation OR Educati* OR method* OR leaflet* OR regulation OR experimental studies (Exact major subject heading) OR nursing process (Exact major subject heading) OR Education (Exact major subject heading) OR professional practice (Exact major subject heading) OR program development (Exact major subject heading) OR rules and regulations (Exact major subject heading) OR government regulations (Exact major subject heading)

**Cochrane Library**
Organ donation OR tissue donation OR organ procurement OR tissue procurement OR eye donation OR cornea donation OR organ transplantation (MeSH descriptor) OR tissue transplantation (MeSH descriptor) OR tissue and organ procurement (MeSH descriptor) OR tissue donors (MeSH descriptor) OR tissue and organ harvesting (MeSH descriptor)<br>AND<br>Healthcare worker OR health employee OR healthcare staff OR health personnel OR healthcare provider OR health professional OR health practitioner OR health manpower OR nurse OR physician OR resident OR health personnel (MeSH descriptor) OR health manpower (MeSH descriptor) OR students, health occupation (MeSH descriptor) OR students, medical (MeSH descriptor)<br>AND<br>Information OR clinical trial OR dissemination OR guideline OR protocol OR policy OR process OR recommendation OR incentive OR organization OR administrati* OR optimi* OR improve* OR project OR workshop OR campaign OR poster OR leaflet OR session OR intervention* OR program* OR strategie* OR practice* OR regulation OR training OR implementation OR program development (MeSH descriptor) OR professional practice (MeSH descriptor) OR education, professional (MeSH descriptor) OR government regulation (MeSH descriptor) OR education, nursing (MeSH descriptor)

**Embase** (1974-2010)
Organ procurement OR tissue procurement OR organ donation OR tissue donation OR organ donor OR tissue donor OR eye donation OR cornea donor OR 'organ donor' (explode) OR 'transplantation' (explode)<br>AND<br>health care workers OR health employees OR health care staff OR health care personnel* OR health care personnel OR health care providers OR health professionals OR health crew OR health teams OR nurse* OR physician* OR resident* OR 'health care personnel' (explode)<br>AND<br>clinical trials OR 'guidelines' (explode) OR protocol* OR 'policy' (explode) OR recommendation* OR incentiv* OR intervention* OR program* OR educati* OR strategie* OR practice* OR training* OR implementation OR campaign* OR workshop* OR session* OR leaflet* OR 'intervention study' (explode) OR 'professional practice' (explode) OR 'nursing intervention' OR 'education program' OR 'program development'

**MEDLINE** (Pubmed:1966-2010)
Organ procurement* OR tissue procurement* OR organ donation* OR tissue donation* OR organ donor* OR tissue donor* OR organ transplant* OR eye donation* OR eye donor* OR corneal donor* OR corneal donation* OR tissue and organ procurement (MeSH major topic)<br>AND<br>health care worker* OR health employe* OR health care staff OR health personnel* OR health care provider* OR health professional* OR (health AND (crew* OR team* OR practitioner* OR worker* OR manpower) OR nurse* OR physician* OR resident* OR health personnel (MeSH major topic) OR students, health occupations (MeSH major topic)<br>AND<br>information* OR clinical trial* OR dissemination* OR guideline* OR protocol* OR policy OR policies OR process* OR recommendation* OR incentive* OR organization* OR administrati* OR optimi* OR improv* OR project* OR workshop* campaign* OR poster* OR leaflet* OR session* OR intervention* OR program* OR strategie* OR practice* OR regulation OR trial* OR training OR implementation OR intervention studies (MeSH major topic) OR nursing process (MeSH major topic) OR program development (MeSH major topic) OR professional practice (MeSH major topic) OR government regulation (MeSH major topic) OR facility regulation and control (MeSH major topic)

**PsycInfo** (1960-2010)
Organ procurement* OR tissue procurement* OR organ donation OR tissue donation OR organ donor* OR tissue donor* OR organ transplant* OR tissue transplant* OR eye donation OR eye donation OR cornea donor* OR tissue donation (index terms) OR organ transplantation (index terms)<br>AND<br>health care worker* OR health employe* OR health care staff OR health personnel OR health care provider* OR health professional* OR (health AND (crew OR staff OR practitioner* OR manpower) OR nurse* OR physician* OR resident* OR health personnel (index terms)<br>AND<br>information* OR clinical trial* OR dissemination OR guideline* OR protocol* OR policy OR policies OR recommendation* OR incentiv* OR optimi* OR improv* OR project* OR intervention* OR program* OR educati* OR strategie* OR practice* OR training OR implementation OR workshop OR campaign OR regulation OR leaflet* OR session* OR group intervention (index terms) OR school based intervention (index terms) OR program development (index terms) OR educational program (index terms) OR personnel training (index terms)

**ProQuest Dissertations and Theses** (1861-2010)
organ procurement OR tissue procurement OR organ donation OR tissue donation<br>AND<br>health care worker OR health professional OR health personnel OR nurses OR physicians<br>AND<br>education OR intervention OR program OR practice OR guideline OR campaign OR session OR workshop OR leaflet OR regulation
